# Contextual Exploration of a New Family Caregiver Support Concept for Geriatric Settings Using a Participatory Health Research Strategy

**DOI:** 10.3390/ijerph14121467

**Published:** 2017-11-28

**Authors:** Elisabeth Dorant, Theresia Krieger

**Affiliations:** Faculty of Health Medicine and Life Sciences, Maastricht University, 6200 MD Maastricht, The Netherlands; Theresia.krieger@maastrichtuniversity.nl

**Keywords:** participatory health research, family caregiver support, geriatric department, engagement, exploration, complex intervention, professional capacity building

## Abstract

Family caregivers are the backbone of the long-term care support system within the home environment. Comprehensive caregiver support programs require collaboration and coordination within the system. A new public health concept, Vade Mecum, aims to harmonize and professionalize family caregiver support initiatives in geriatric care settings in the Euregion Maas-Rhine. Exploration of the new concept recently started in Germany to gain in-depth insight into current support and the needs of the geriatric care team and family caregivers. Within the context of an exploratory qualitative study, a participatory health research (PHR) strategy was applied to make optimal use of experience and knowledge from the system. Care professionals, engaged as co-researchers, were responsible for decisions about the research question, data collection methods and procedures of engaging family caregivers. A research team representing all professions within the geriatric department was formed. Research objectives were formulated and an appropriate mix of qualitative data collection methods consisting of interviews, focus groups and story-telling was chosen. Needs and expectations of the new concept, and practical solutions for involving family caregivers were discussed. A PHR strategy resulted in initiating a qualitative study in a geriatric care setting carried out by care professionals from the department. Knowledge was generated in a co-creative manner, and co-researchers were empowered. A comprehensive understanding of the system serves as a starting point for advancement of the new family caregiver concept.

## 1. Introduction

A vast majority of Europeans prefer to be cared for in their own home or the home of their family when in need of long-term care [1]. Family caregivers are the backbone of the long-term care support system. Family or informal caregiving is a “free of charge” service, provided by a relative, partner, friend or neighbor to a person with a chronic disability [2].

Recent figures show that in Germany, 69% of all people requiring long-term care are cared for at home, with over 90% receiving care from family members. More than 70% of home care is provided by family members alone, without the assistance of care professionals [3]. Elderly people, especially rely on their family for day-to-day care or support. In Belgium, almost 10% of persons aged 15 or over provide informal care, with the most intensive informal care (more than 4 h per day) concentrated in older age groups (65+), most of who care for their spouse [4]. In The Netherlands, more than 50% of all adults over 75 years of age living in the community receive care and support; 22% receive care only from a family caregiver [5].

Although caregiving can have positive consequences, there is ample evidence that family caregiving has a negative impact on the physical, mental and social well-being of the caregiver [6,7,8,9]. The scale of these consequences is related to both the patient’s condition and the amount of time needed for their care, as well as to their own needs. The caregiver’s characteristics, such as their age, health, being employed, and the presence of resources, influence the impact of caregiving [6,7,10].

Caregivers can feel overburdened for different reasons, for instance, to be “24 h on call”, or to have to manage the bureaucratic work that is involved to get care organized and running [6,11]. When family caregivers are not involved with care professionals as partners in the patient care trajectory they may not have sufficient knowledge about practical aspects of the care that has to be provided [6]. They may also experience that formal health care providers give little structured guidance to them regarding their prospective role as caregiver [12,13,14], or that professional caregivers do not have enough understanding regarding the next of kin’s worries and stresses about caregiving [15].

Caregivers of older adults experience burdens different from those of other caregiver populations [16]. Problem behavior of elderly care recipients may be the underlying source of the increased burden [7]. Also, the high prevalence of multi-morbidity among elderly persons may influence the complexity of the role of caregivers in the home environment [17], and thus increase the support needs of their family caregivers [18].

Family caregiving for elderly persons at home may be especially demanding in situations when the family member who is taking up the role as caregiver has no experience, or when there is a sudden demand. They may feel unprepared for their role [6,19]. In these situations, the caregiver, at the same time, has to be prepared for the practical aspects of their caregiving task and to deal with the unexpected emotional strains resulting from the care receiver’s condition. Family caregivers need personalized information, psychological support, effective communication and financial and legal support [20,21]. Timing of the support and the way of approaching caregivers is crucial [22]. Long-term support and outreach counselling may be needed [23]. Remarkably, however, the overall effect of current tailored caregiver support programs for elderly living in the community seems to be small, as was concluded in a recent systematic literature review. This disappointing outcome was attributed to inconsistencies between the included studies, but the main recommendation was that support for family caregivers of the elderly requires intense collaboration and coordination between all stakeholders [24]. 

**Figure 1:**
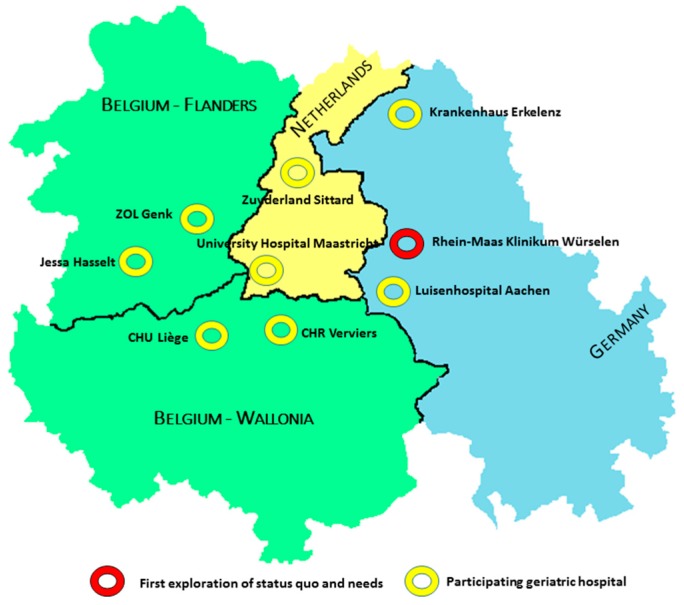
Geographic cross-border orientation of the recently initiated family caregiver support program (Vade Mecum): hospitals in three countries in the Euregion Maas-Rhine joining the project. ZOL: Ziekenhuis Oost-Limburg Genk; CHU: Centre Hospitalier Universitaire de Liège; CHR: Centre Hospitalier Regional Verviers.

In this article we describe an initial step in the advancement of a new and innovative family caregiver support concept for elderly persons called Vade Mecum. Vade Mecum is a recently initiated complex public health intervention specially designed to harmonize and professionalize current caregiver support initiatives in geriatric care situations, with special attention to cross-border continuity of support. Vade Mecum is situated in the Euregion Maas-Rhine (EMR), an area where three countries (Germany, Belgium, and The Netherlands) are closely connected and health services operate cross-border. This specific geographical configuration is adding to the complexity of family caregiver support in the case of cross-border hospitalization or rehabilitation of the care receiver. Nine hospitals with geriatric departments spread over the EMR participate in the project (Figure 1).

The new and innovative Vade Mecum concept builds on prior knowledge and experience from a recent innovation project, The Caregivers’ Guide, a professional stroke caregiver support program executed from 2012 to 2015 in Aachen, Germany [25]. The Caregivers’ Guide concept consists of eight conceptual building blocks: five core building blocks encompassing an individualized caregiver counselling program and three facilitating building blocks safeguarding and interconnecting the program with the practical, real-world, system. The Caregivers’ Guide offers a specially trained counsellor who guides the family caregiver through the entire stroke trajectory of the care receiver and provides tailored family caregiver support from as early as possible after a stroke to as long as needed in the home environment. The concept of the Caregivers’ Guide was aligned with the real-world system before implementation using participative action research. The program’s stakeholders played a crucial role in this process [26,27].

The new Vade Mecum concept is based on the earlier Caregiver’s Guide concept. In the Vade Mecum project, care professionals employed in one of the hospitals joining the project will receive special training to become a specialized Geriatric Family Companion, a new job profile as a hospital-based family caregiver support specialist. As a group, the Geriatric Family Companions will form a cross-border network of support experts, offering personalized counselling, information provision, empowerment and resource allocation to first-time, informal caregiver–geriatric patient dyads. Family caregiver support will start in the hospital, as early as possible after hospitalization of the care receiver, and will last until at least a secure situation in the home environment is reached. The ultimate objective of the new Vade Mecum concept aims to sustain health and wellbeing, and prevent social exclusion and health inequality of geriatric patient-caregiver dyads. The focus of the primary prevention support program is on the family caregiver.

Early engagement of the system in which Vade Mecum will be operating, is paramount to achieve the goals of this new complex public health intervention. The Meikirch model of Health will serve as a framework to keep the focus on the interconnectedness in the system [28]. The management of the entire project as well as all research associated with it, is guided by tools from project management and a design thinking approach [29,30]. The project is divided into five consecutive steps: (1) 360° exploration; (2) concept and curriculum development; (3) training of Geriatric Family Companions; (4) implementation and supervision; and (5) evaluation.

For the first 360° exploration, the participatory health research (PHR) paradigm was considered as most appropriate, since it involves exploration of local knowledge and perceptions from key stakeholders who are affected by the intervention or provide a service to the family caregivers [31,32]. PHR, which has its roots in participatory action research, adult education, medical anthropology, agricultural and community development [31], is a research strategy broadly defined as “systematic inquiry, with the collaboration of those affected by the issue being studied, for purposes of education and taking action or effecting change” [33]. The bottom-up PHR strategy is characterized by the reflective and open-minded attitude of the research team and an open process-oriented outcome [34]. It targets two goals: co-creation of knowledge with respect to practical questions and empowerment of the co-researchers [31,35].

In this article we describe the initial step in the development of a new holistic family caregiver concept applying a PHR strategy. The results of this first exploration will serve as starting point for further concept development.

## 2. Materials and Methods

### 2.1. Research Design and Strategy

**Figure 2:**
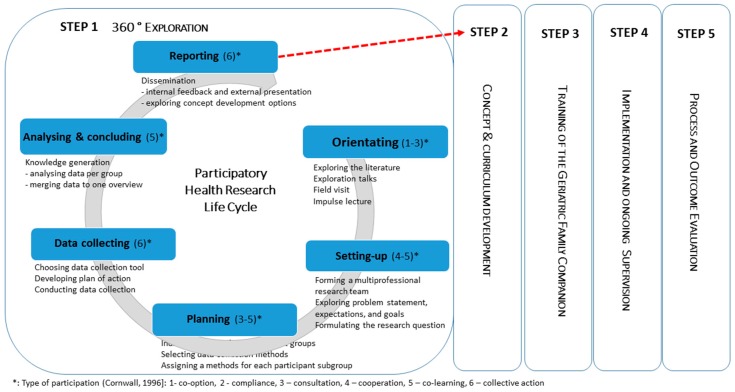
Project management life cycle and type of participation in the early 360° exploration of the new Vade Mecum concept.

Within the context of the first step in the development of the new Vade Mecum concept, the qualitative 360° exploration of the system, a stepwise PHR strategy was applied to gain a deeper understanding of the current family caregiver support system and of the support offers and needs in the geriatric setting (see Figure 2).

PHR enables proactive stakeholder involvement and empowerment. “Information rich” stakeholder groups who have a depth of experience towards family caregiver issues in a geriatric setting contribute to the research process, thus ensuring that the research is conducted not just on, for and with people, but also by people from this setting [31]. Our PHR strategy will be restricted to practicable, qualitative data collection methods in order to enrich the geriatric family caregiver support concept. By drawing from the breadth of expertise and knowledge in the participating geriatric care system, the description of the new Geriatric Family Companion’s job profile will be based on the actual needs of the setting in which the new job will operate.

### 2.2. Setting and Research Team

PHR started in February 2017 in the Rhein-Maas-Klinikum (formerly known as Medizinisches Zentrum Würselen), Germany. Since 1998 this hospital has had a geriatric department. Today it contains 64 acute geriatric beds and 28 rehabilitation beds spread over three wards. The department has a very good reputation within the community.

Presently, the multidisciplinary professional team on the geriatric department consists of more than 70 members: 10 medical doctors, 44 nurses, 12 therapists (ergo- and physio-), 2–3 neuropsychologists, 2 case managers, and 2 social workers. Care and medical support is provided with a patient-centered approach (professional–patient dialog). The department is characterized by a low staff turnover.

In 2016, 1550 patients, on average 82.7 years old, were admitted at the geriatric department with diagnoses varying from heart and vascular disease to neurologic disorders, severe accidents, pneumonia, Parkinson’s disease, and hip fractures. Co-morbidity with dementia or delirium was estimated at 50%. After a mean stay of 17.8 days, about half of the patients returned home where approximately 60–70% needed daily assistance. The re-hospitalization rate within 3 months after hospital discharge is estimated at 10%.

Members of the multi-professional care team working at the geriatric department, as well as professionals with a broader perspective working elsewhere in the hospital will be engaged as co-researchers. The research project is supported by two external researchers: (1) a critical friend with experience as a nurse and health manager; and (2) an academic researcher facilitating and stimulating the research process and providing the scientific and technical knowledge.

### 2.3. Project Realization and Data Collection

The project life cycle for this early 360° exploration contains six consecutive phases: (1) orientation; (2) setting-up; (3) planning; (4) data collecting; (5) analyzing and concluding; and (6) reporting (Figure 2).

Care professionals will be engaged as co-researchers in all phases of the study. They will define the research problem and the project goals, formulate the research question, choose the methods of data collection, conduct the research and analyze the data. The type of participation of the co-researchers, defined using an adaptation of Cornwall’s participation model (see Table 1, [36]), will change through the process of active engagement and empowerment while the project is progressing.

The perceptions, insights and experiences of the professional care team (as service provider), the family caregivers (as service receiver) and a broader perspective from the co-researchers working in the hospital will create the projects’ knowledge base. To enable triangulation, a mix of different qualitative participatory data collection and analysis methods will be used.

## 3. Results

Results of this ongoing PHR study will be presented for the first four phases of the six-phase PHR life cycle (see Figure 2).

### 3.1. Phase 1: Orientation—March 2017

The orientation phase started with a brief inspection of the literature to gain insight into the current state of knowledge concerning caregiver support initiatives in geriatric settings.

Next, two explorative conversations with the head of department were held by the external researchers followed by a two-day field visit (Theresia Krieger, second author), both with special focus on family caregiver support. Initial personal impressions were that care professionals perceive family caregivers as important for the rehabilitation process, but that their role was not specified within the current rehabilitation process. Support offers were perceived as fragmented, uncoordinated and lacking a holistic approach. A “trialogue”, involving caregivers as partners in the care process, was offered on request but was principally patient-centered. Caregiver burdens or needs were not assessed systematically. However, family caregivers might have received some support within the system from different professionals on the department, e.g., nurses, physiotherapists; or from external support providers, e.g., from communal social services (Pflegestützpunkt) if they requested this support.

Next, an impulse lecture was offered by the external researcher, where the idea of conducting a PHR project with the focus on family caregivers of geriatric patients was presented to members of the current multidisciplinary care team as prospective co-researchers. The professionals present at this first meeting were actively invited to take part in the project as a co-researcher. Nine professionals expressed their interest spontaneously and joined the research team. To further expand the team, and to make sure that all professions would be represented in the research team, a form for completion was placed centrally in all three wards for three weeks, accompanied by a flyer.

### 3.2. Phase 2: Setting Up—April 2017

The second phase started with setting up the project research team. Eventually, the research team consisted of 16 members: medical doctors (*n* = 2), case managers (*n* = 2), social workers (*n* = 2), therapists (physiotherapy, logotherapist) (*n* = 2), and nurses (*n* = 4). Moreover, one nurse teacher from the nursing school, and the pastor working for the geriatric department decided to join the research team. The two external researchers facilitated the research process. All team members were experienced in their own field, expressed open-mindedness and interest in the topic and the participatory working approach.

In the first workshop the research team contemplated: (1) the problem statement; (2) the goals and expectations concerning the family caregiver support facility; and (3) the research question.

**Figure 3:**
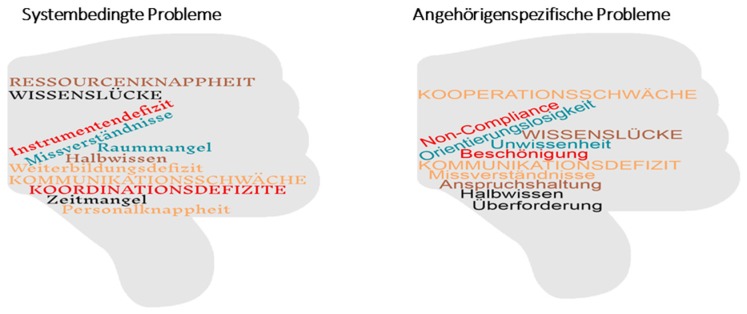
System and caregiver problem identification in the geriatric setting: word clouds as presented to the research team (German language).

(1) Problem definition. Using the brainstorming method, all team members reflected upon their daily challenges in providing caregiver support. This interactive process resulted in formulating the problem statement. System-related problems, such as knowledge gaps, lack of resources, coordination deficits, as well as caregiver-related problems, such as non-compliance, overburdening, and communication deficits, were identified. Important individual contributions from members of the research team concerning potential problems were combined in a word cloud and presented as an overview to the research team (Figure 3).
(2) Expectations. The expectations concerning the new job description of the Geriatric Family Companion were explored by the professionals as a group as well as for each individual team member personally, and for the caregiver–patient dyad as perceived by the care professionals. Discussions took place in small groups of 3–4 professionals (“Murmelgruppen”) after which the results were presented to the entire research team. The team then structured and prioritized the findings. Individual care professionals expect to: have more time personally to support caregivers individually according to their different needs when support is scheduled in the normal working hours and not in extra time, benefit from task simplification, make personal improvements and get appreciation. For their own profession, they also expect improved time allocation, a better infrastructure, and improved competence. The project might help to give caregivers and patients more consultation time, individualized and improved support, as well as more satisfaction with the services.

Expectations were listed per group in a table, which was sent to the entire research team after the workshop (Table 2).

(3) Research question. As most participants indicated they were inexperienced in formulating a research question, they asked for moderation by the external researchers. Triggering questions such as: Who is the focus of interest? What do we want to know? Where does the study take place? helped the research team in formulating the research question: “How does the multidisciplinary research team of the Rhein-Maas Klinikum currently support family caregivers, and what is needed to provide tailored support to caregivers?”

### 3.3. Phase 3: Planning—May/June 2017

In the planning phase, first a decision had to be made concerning data collection methods suitable for the care professionals as service providers, and for the caregivers as service receivers. The team also had to decide on the timing of the actual data collection within their respective settings.

At the start of the second workshop the external researcher briefly presented five frequently used qualitative data collection methods, addressing their advantages and disadvantages: interview, focus group, structured interview matrix [37], story-telling [38], and community mapping [39]. A mix of different data collection methods was recommended to enable triangulation. Next, the presented data collection options were discussed with regard to their feasibility and potential for knowledge generation for each professional group. Since family caregivers were not included yet in this planning phase of the PHR process, it was contemplated who best to approach and which data collection method would be suitable to use. One of the nurses proposed to invite “returners”, i.e., family caregivers with experience as a caregiver to the geriatric ward, to join in a story-telling activity. The social worker suggested that new, i.e., first-time, caregivers may be open for an interview. The group agreed with these ideas.

**Figure 4:**
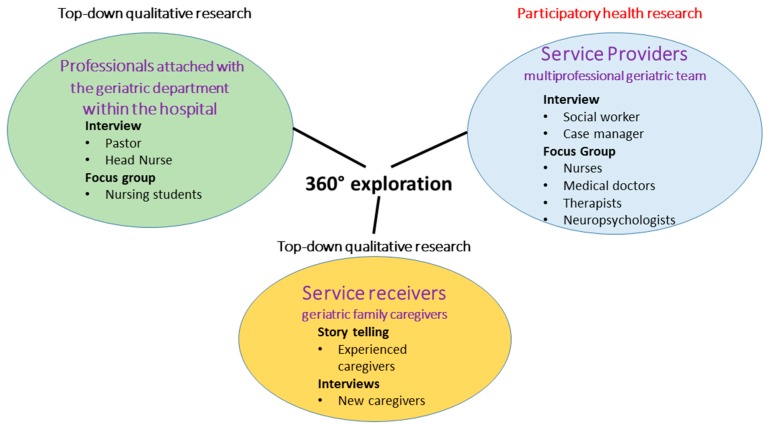
Qualitative data collection methods per participating professional group.

Finally, it was decided by the entire research group to use interviews, focus groups, and story-telling to generate new knowledge (Figure 4).

In a third workshop (June 2017) the timing of data collection was scheduled, responsibilities were clarified within the geriatric department, and a plan of action was drawn. All information was inserted in a Gantt chart and distributed to the entire research team. An example of this chart is provided in Table 3.

It became apparent that it was necessary to structure, guide and synthesize the process of data collection, and to empower the co-researchers to gather the data. For this a “question catalogue” including 48 questions was developed by the research team and the external researchers, based on the problem analysis in the initial phase of the PHR. It includes the following themes: caregiver needs, information, expertise, skills, resources, management, mandate, and support offers, in 24 status quo and 24 needs questions (see Appendix A, Table A1). This instrument can be used by each profession and adapted to the needs of the group of participants when conducting interviews, focus groups, and storytelling. The questions were tested for their general understanding in a team discussion led by the external researcher. The external researcher also provided a checklist for preparing and conducting interviews and focus groups in a participatory manner. One professional group (case management) needed further explanation before starting their data collection process, the other groups felt empowered by these two instruments. 

For recruiting the service receiver group a flyer was developed and distributed to potential caregivers within the geriatric department.

### 3.4. Phase 4: Data Collection—June/September 2017

The participatory data collection process is planned to be conducted by the co-researchers between June and September 2017 (Table 2). The family caregiver perspective will be gained in September 2017. The perspective beyond the geriatric ward (nursing students, pastor, and head nurse) will be explored by the external research researcher between June and September 2017.

### 3.5. Phase 5: Data Analyses and Conclusion—October/November 2017

For this part of the research project co-researchers will be assisted by the critical friend and the external research researcher. Qualitative data will be analyzed using thematic content analysis.

### 3.6. Phase 6: Reporting—December 2017

Finally, in the last phase findings will be disseminated internally by members of the research team within their own geriatric setting, and externally, e.g., through scientific publications or at conferences.

## 4. Discussion

Two important goals were reached in this first exploration of the current family caregiver support system in the German geriatric setting applying a PHR strategy: knowledge was generated in a co-creative manner, and co-researchers were empowered within their setting. In PHR, researchers are assigned to two roles: facilitators and learners [31]. In our 360° exploration the two researchers, both external researchers, supported the co-researchers to grow in their new role, build up confidence as well as knowledge to be able to conduct PHR, and facilitated, structured and guided the entire process.

In our study, the application of PHR as a research strategy had several advantages: the problem was illuminated from different perspectives, research objectives were developed from the real-life experiences of professionals within the setting, and data collection methods were chosen on the basis of feasibility and the requirements within the setting. Also, co-researchers came up with practical solutions for unforeseen problems (e.g., how to approach the caregivers). PHR gave us insight into local practices and possibilities for change.

PHR concentrates on knowledge for action with emphasis on a bottom-up approach [31]. Participation requires reflexive stakeholder engagement, which can help identify problems, and systematically implement, monitor and reflect the outcome of change [40]. PHR must be understood as a process, requiring time to build up relationships and trust, which are necessary to work in a creative way. Investing in communication and information sharing is a key requirement for success [31,32]. Throughout the course of these first steps in our project’s life cycle we were able to increase the level of participation from consultation to co-learning [36].

A deep participatory process engages different participants in all stages of a given activity, from identification to decision-making. However, this is time consuming as we experienced in our PHR project. Since our approach was used for the first time in this geriatric department, the newly appointed co-researchers needed time to adapt to their role.

Compared with conventional top-down research we also experienced that PHR requires an extra time investment from the researcher’s side [31]. The co-researchers also needed to invest considerable time, which was described by some as “on top of the other work”, as the research activities were not perceived as a priority in the department.

PHR also requires the ability for critical reflection and knowledge about research methods. Since the majority of the co-researchers had no experience and were not involved or trained earlier in qualitative research methods, they needed extra guidance and facilitation by the external researcher.

The open-ended, less controllable outcomes typical of a PHR project, may have tested some participants as well. However, in our case the co-researchers did not feel stressed by the chosen methodology of the study and could work with an open mind. As the decision process in PHR is democratic, team dynamics can appear to be challenging as well [41].

During our research, we took the validity criteria of the International Collaboration for Participatory Health Research as guidance, acknowledging participatory, intersubjective, contextual, catalytically, ethical, and empathic validity [32].

We decided to start in one of the larger hospitals participating in the Vade Mecum project by consulting those people, i.e., key stakeholders, who work with and experience the family caregiver–geriatric patient dyad on a daily basis. We felt that it is important to obtain the commitment of the organization’s management although this was only partly achieved in this project. Despite multiple attempts, the head of nurses could not be involved in the study. Overall, establishing a stable research team in a setting of scarce resources took perseverance. Nevertheless, we were able to show that starting and applying a PHR methodology was feasible in the hierarchical setting of the participating hospital.

We have chosen to show, in detail, the outcomes of a first explorative step using PHR in the advancement of a new and innovative, complex public health intervention, Vade Mecum. In our view, it demonstrates the feasibility and value of taking a systems approach and include key stakeholders as collaborative research partners. Recent examples of research applying similar methodologies show the potential benefits of starting with a participatory methodology in the exploration phase of a new (public) health intervention. For instance, a family support strategy for family members of people with traumatic brain injury was developed using participatory methods [42]. In their article the researchers provide a thorough description of the participatory process they used to develop their program by involving clinical teams, hospital management teams, and a large number of client-family groups. Co-creation resulted in an eight-tiered approach to change support practices for family members [42]. In another innovative project about inpatient mental health services for young people with psychosis admitted to a mental hospital, a detailed description was given of the successful identification of areas for service improvement by incorporating practical knowledge and the expertise of service users, caregivers, community, and inpatient staff, as well as management [43].

While planning the development, implementation and evaluation of our new complex geriatric caregiver support concept in the EMR, we acknowledged the importance of contextual exploration as a distinct first step in the process of service development, as it allows requirements and specifications to emerge [44]. The Meikirch model we chose as guidance enabled us to deconstruct and make sense of the different levels of the health care system around the family caregiver–geriatric patient dyad, and to be aware, beforehand, of the potential interactions between the different levels [28].

Recommendations for first explorative steps in the development of new complex interventions in public health sometimes lack a focus on systems thinking. Prominent guidelines on development of complex interventions such as the Medical Research Council UK (MRC) [45], or textbooks on the subject, e.g., [46], primarily build on knowledge from evidence-based medicine, and focus almost entirely on the translation of already tested, mostly under highly controlled conditions, interventions for implementation in public health settings. However, conventional approaches commonly used in medical research to design and evaluate interventions may not be advanced enough to understand the context and connections between the parts, the actors and the processes of the system [31,47]. Integration of expertise from the field is crucial to the success of innovation [48], and collaboration with key stakeholders, forming joint and equal working partnerships, is critical to build the political context in which the project will develop [49]. Partnerships with stakeholders can also bring alternative perspectives, as we experienced in our project. Stakeholders may have personal skills, knowledge, experiences and abilities that complement the expertise of the researcher, which can contribute in the divergent generation phase of concept development as well as in the convergent selection phase of new interventions [41].

We decided to apply conventional project management tools for operational planning and control, in combination with a design thinking approach as an innovation strategy. Traditionally, project management is a performance oriented practice aiming at the constitution, coordination, and control of activities within a project [50]. Design thinking refers to a human-centered approach to innovation that puts the observation and discovery of human needs at the forefront of an innovation process and starts with observing the users and the system’s context and constraints [30]. Design thinking, with its emphasis on learning and knowledge creation, can be viewed as a novel methodology and a potentially valuable practice for improving innovation outcomes, whether those outcomes are products, services, or strategies [41]. Combining project management with a design thinking approach can provide significant contributions with respect to problem as well as solution formulation encountered in complex projects [41,51].

### Considerations

The composition and social dynamics of the team engaged in PHR may have influenced the results of this exploratory investigation. It is not clear to what extent reflections on the group’s objectives, strategies and processes took place and how that influenced the outcomes of their task. Also, it is uncertain if this led to a different perception of the problem or a different problem statement. Furthermore, participants in the system, such as our participating hospital, need adequate resources, also in the long-term, to be able to make sustainable changes. They also need to build capacity to deliver the intervention. Time, effort and resources, and staff skill development need to be secured, which may be a challenge. During the research process a stable team without member turnover, as well as organizational commitment is required to maintain effective research relationships. Mutual respect and a working partnership, effective communication, financial support and accessible information are important ingredients for continued stakeholder engagement [52].

Further challenges in the future of the Vade Mecum project will be the identification and engagement of other or new relevant stakeholders to sustain the realization of project and advance the large and complex Vade Mecum project. However, we are encouraged by this first investigation using PHR and feel that this approach will lead to in-depth contextual understanding and engagement of all stakeholders before moving to the next phase of Vade Mecum, the concept and curriculum development.

## 5. Conclusions

During this first study in applying a PHR strategy we achieved a high degree of participation, our co-researchers were empowered, and new knowledge was generated in a co-creative manner. In this early explorative step in the development of an innovative family caregiver support concept, individuals of the multidisciplinary geriatric care team became co-researchers; the problem statement, goals and expectations as well as the research question were formulated; practical solutions for getting access to the support receivers were found; and suitable data collection methods were chosen in a democratic way. In forthcoming steps in the current PHR process, data will be collected by the co-researchers, analyses will be conducted by the entire research team, and the co-researchers will be empowered to disseminate their findings.

## Figures and Tables

**Table 1:** Participation type, stakeholder involvement and research relationship. Adapted from [36].

Participation Type	Character of Stakeholder Involvement	Relationship (Researcher and Stakeholder)
1. Co-option	Token; representatives are chosen, but no real action	On
2. Compliance	Tasks are assigned, with incentives; researchers decide agenda and direct the process	For
3. Consultation	Stakeholders’ opinions are asked, researchers analyze and decide on a course of action	For/with
4. Cooperation	Stakeholders work together with researchers to determine priorities; responsibility remains with researchers for directing the process	With
5. Co-learning	Stakeholders and researchers share their knowledge to create new understanding, and work together to from action plans with researcher facilitation	With/by
6. Collective action	Stakeholders set their own agenda and mobilize to carry it out, in the absence of outside researchers or facilitators	By

**Table 2:** Overview of personal and group expectations of participating in the participatory health research (PHR) project.

**Service provider as individual co-researcher**	Time	To provide the adequate caregiver support in the time it needs To do no over-time, when supporting caregivers.
	Personal development	To improve skills in communication, stress and conflict management To develop further social skill (empathy).
	Individual appreciation	To gaining societal appreciation for working in the geriatric ward To do something to improve the situation To foster the individual awareness and intrinsic motivation.
**Service providers as professional team**	Time	To understand the counselling needs of different caregivers To offer structured support (individual information, empowerment).
	Resources	Infrastructural resources (counselling room) Adequately trained and sufficient staff.
	Professional improvements (knowledge, skills)	Communication skills Practical skills transmission to informal caregivers Capacity building for all staff focusing on caregiving and home care Structural understanding and concept development.
	Communication	To improve the work within the multi-professional team To improve the interdisciplinary teamwork To foster the professional image To improve the public image.
	Structured caregiver support (concept)	Early caregiver needs assessment Professional caregiver support focus person Synergize and structure individualised support Caregiver guideline (clarified responsibilities) Structured information flow.
	Obtain professional satisfaction	To experience the professional efficiency when supporting caregivers.
Service receiver * (family caregiver)	Time	To understand the process of the rehabilitation timely planning to get clarification on own responsibilities To identify the right person in charge in the different settings (acute-, rehabilitation-, home care).
	Individualized and improved support	Psychosocial preparation for the new role Early inclusion in planning and treatment processes Training regarding the key competences of informal caregiving.
	Resources	Adequate counselling facility and fixed counselling hours.
	Communication improvements	Personalized information and information material (flyer informing about patient care trajectory) Clarification on medical terminologies.
	Satisfaction	Due to adequate, empathic, and professional support.

* As perceived by the multi-professional team.

**Table 3:** Gantt chart presenting the data collection process in the geriatric department in 2017.

	Year 2017														
Month	June		July				August					September			
Week	25	26	27	28	29	30	31	32	33	34	35	36	37	38	39
**Service provider (multidisciplinary team)**															
Medical doctors							FG								
Nurses											FG				
Therapists (physio/ergo/speech)											FG				
Neuro-psychologist															
Social workers										I					
Case managers										I					
**Service receiver (family caregiver)**															
Experienced caregivers												ST			
New caregivers												I			
**Broader perspective (hospital-intern)**															
Nursing students	FG														
Pastor	I														
Head nurse												I			

FG: Focus group; I: Interview; ST: Storytelling.

## Appendix A

**Table A1:** Question catalogue for the Participatory Health Research project in the Geriatric Department.

Status-Quo Assessment (“How Do You Experience the Current Support?”)	Categories	Needs Assessment (“What Would Be Needed?”)
How do you experience the current family caregiver support in your department? On which occasions do you have contact with family caregivers? Can you recall a meeting with family caregivers?	Warming up	What do you need in general to provide family caregiver support? Which specific support offers would be helpful or applicable in your department?
What burdens family caregivers of geriatric patients? How do you currently meet the family caregivers’ needs? In what situations or circumstances require family caregivers your support?	Caregiver needs	In what situation and circumstances would family caregivers need support?
What type of information do you already have about the family caregivers? Which caregiver-specific information is systematically recorded?	Information about the caregiver	Which information should be systematically recorded in order to enable personalized caregiver support? Would it be helpful if family caregivers will list their needs in a self-registered assessment? Please justify your decision.
What expertise of your own do you use when supporting family caregivers? What expertise and knowledge do you lack? In which caregiver-specific capacity building activities did you participate? Which caregiver-specific capacity building activities are made available for you by your employer?	Expertise (e.g., for example about financing, housing adaptation, clinical picture)	What kind of knowledge would be necessary to advise family caregivers of geriatric patients? What aspects of family caregiver support should be incorporated in education or academic studies? Which further capacity building offers do you require?
Do you feel prepared for dealing with family caregiver issues? What skills do you use in your daily work with family caregivers of geriatric patients? Which skills do you miss? How do you handle "difficult" family caregivers?	Skills (e.g., dealing with anger, grief, lack of compliance, or conflicts)	What skills should you require for family caregiver support? What skills would you like to develop further? What should your employer do to improve your caregiver support skills?
Which resources (infrastructure, time and material) are available for you to provide personalized family caregivers support? Who is in general responsible for providing caregiver support? Why?	Resources (e.g., consultation room, time, counselling material)	Which resources do you require to provide personalized family caregivers support? Would an assessment instrument for family caregivers make sense? Please explain! What advantages would a focus-person system for family caregivers imply for: (a) family caregivers, (b) patients, (c) geriatric support team, and (d) support system in the home care environment?
How would you define your role in the current family caregiver support process? Are there designated office hours available for family caregiver support?	Management (“How do you engage with your family caregivers?”)	What should be your role in family caregiver support in the future? How should caregiver support offers be managed in your department?
To which extent is supporting family caregivers outlined in your job description, or do you feel ‘morally’ committed? How would you estimate the level of importance of family caregiver support in the eyes of your boss?	Mandate	What should your hospital management do to enable and maintain personalized family caregiver support? How could your daily support efforts be more appreciated by the management?
Which specific family caregiver support offers exist, or existed, within your setting and are/were these offers accredited? Which external offers (e.g., from the communal services) do you know? And which do you recommend, and when? Where do you spot weaknesses in the current caregiver support?	Family caregiver support offers (“What are important ingredients for a personalized caregiver support?”)	What should the ideal support offer for family caregivers in your department look like? Please reflect on the following items: Counselling type: personal counselling/group/mixed Informational support: personal conversation/flyer/internet/mixed Approach: active research for caregivers on the department/relatives come to office hours Setting: in the department/home environment/flexible Funding: hospital, municipality, health insurance, own contribution, mixed.

Instruction: Co-researchers from the multidisciplinary geriatric team (nurses, medical doctors, therapists, social workers, and case managers) can select from each category listed in the catalogue the most suitable question(s). Please, choose questions from the ‘status-quo assessment’ as well as from the ‘needs assessment’ list according to their applicability in your own profession/situation to guide interviews and focus groups among your peers. Take note of the research question: “How does the multidisciplinary research team of the Rhein-Maas Klinikum currently support family caregivers, and what is needed to provide tailored support to caregivers?”.
